# “The eyes are the windows of the soul”: Portable automated pupillometry to monitor autonomic nervous activity in CO_2_ narcosis: A case report

**DOI:** 10.1097/MD.0000000000033768

**Published:** 2023-05-12

**Authors:** Junko Yamaguchi, Kosaku Kinoshita, Toru Hosokawa, Shingo Ihara

**Affiliations:** a Division of Emergency and Critical Care Medicine, Department of Acute Medicine, Nihon University School of Medicine, Tokyo, Japan.

**Keywords:** autonomic nervous activity, case report, CO_2_ narcosis, miosis, pupillometry

## Abstract

Altered autonomic responses can be observed in patients in the intensive care unit (ICU), and these changes in abnormal autonomic responses are known to be associated with patient prognosis. Therefore, it is important to monitor autonomic nervous system activity in these critically ill patients. While the utility of monitoring critically ill patients using heart rate (HR) variability measurements has been reported, portable automated pupillometers are small, lightweight, and easy-to-operate medical devices that may be more easily evaluated for autonomic nervous system function.

**Patient concerns:**

An unconscious 80-year-old female patient with chronic obstructive pulmonary disease was brought to the medical emergency department after a call from her caregiver.

**Diagnosis:**

On arrival, the patient’s Glasgow coma scale score was 7, her blood pressure was 140/80 mm Hg, her HR was 114 bpm, and her respiratory rate was 27 breaths/minutes with increased breathing effort. Oxygen saturation was 90% on a venturi mask (3 L of supplemental oxygen). The arterial blood gas analysis showed a pH of 7.196, a partial pressure of carbon dioxide (CO_2_) of 89.6 mm Hg, a partial pressure of oxygen of 87.5 mm Hg, and a bicarbonate level of 29.4 mmol/L. Other than CO_2_ narcosis, there were no abnormal findings to induce impaired consciousness. The patient did not respond to support with a bag-valve mask and was intubated. One hour after intubation, her impaired consciousness improved. The patient was extubated 20 hours later and discharged on Day 3.

**Interventions:**

The patient was admitted to the ICU after being intubated, where vital signs and blood gas analysis were monitored every 2 hours, and consciousness was assessed using the Glasgow coma scale. Using a portable automated pupillometer (NeurOptics NPi™-200, Neuroptics Inc., Irvine, CA), pupillary responses, including pupil size or light reflex, were measured every 2 hours during ICU stay.

**Outcomes:**

Changes in respiratory rate and partial pressure of CO_2_ values correlated with pupil size and constriction velocity, but HR changes were contrary.

**Lessons:**

Pupillary responses exhibited by automated pupillometers observed in patients with CO_2_ narcosis may be linked to vital signs and allow for autonomic evaluation.

## 1. Introduction

Changes in autonomic nervous system (AN) activity in patients in the intensive care unit (ICU) are known to be associated with their prognosis and pathophysiology,^[1]^ and pathophysiological analysis based on the assessment of AN function for these patients is crucial to improve outcomes. The utility of monitoring critically ill patients using heart rate variability (HRV) measurements has been reported.^[1,2]^ Portable automated pupilometers are small, lightweight medical devices that are easy-to operate. In recent years, several reports have indicated the possibility of using pupilometers for complete, accurate assessment of neurological monitoring due to better correlations between pupilometer measurements and changes in intracranial pressure in patients with central nervous system diseases.^[3]^ The pupils are regulated by the AN, similar to vital signs, such as heart rate (HR), respiratory rate (RR), and tidal volume (TV).^[4]^ However, the results of the correlation of pupil size with RR and TV were obtained from volunteers in an experimental study.^[5]^

Regarding carbon dioxide (CO_2_) narcosis, portable automated pupillometry monitoring may make it possible to easily assess the patient’s AN activity. In this case, we investigated the correlation between pupillary responses and vital signs to evaluate whether portable automated pupillometry is a useful tool to assess AN activity.

## 2. Case report

### 2.1. Patient information

An unconscious 80-year-old female patient with chronic obstructive pulmonary disease (COPD) was brought to the medical emergency department after a call from her caregiver. She was admitted to the emergency room (ER) due to impaired consciousness caused by CO_2_ narcosis and was hospitalized for treatment 3 months prior. She had neither familial/psychosocial history nor any autonomic nervous dysfunction diseases.

### 2.2. Clinical findings, timeline, and diagnostic assessment

On arrival at the ER, she had a conscious disturbance and had a Glasgow coma scale (GCS) score of 9 (E1V1M6). The patient’s blood pressure was 143/75 mm Hg, her HR was 113 bpm, and her RR was 27 breaths/minutes with increased breathing effort. Oxygen saturation was 90% on a venturi mask (3 L of supplemental oxygen). The arterial blood gas analysis showed a pH of 7.196, a partial pressure of carbon dioxide (PCO_2_) of 89.6 mm Hg, a partial pressure of oxygen of 87.5 mm Hg, and a bicarbonate level of 29.4 mmol/L. The patient was intubated but did not respond to support with a bag-valve mask. Her impaired consciousness level and respiratory effort improved. Although she took fentanyl, midazolam, and rocuronium bromide intravenously for intubation at the ER within 1 hour, her consciousness level improved only 1 hour after intubation, from a GCS score of 7 (E2V1M4) to a GCS score of 11 (E4VtM6). Other than CO_2_ narcosis, there were no metabolic abnormalities that would cause impaired consciousness or abnormalities on head computed tomography evaluation. In addition, she had previously experienced a CO_2_ narcotic episode. We diagnosed the cause of impaired consciousness depending on the attribution of exacerbations of COPD.

### 2.3. Interventions

The patient was admitted to the ICU after being intubated; here, vital signs and blood gas analysis were monitored every 2 hours, and consciousness was assessed using the GCS. Using a portable automated pupillometer (NeurOptics NPi™-200, Neuroptics Inc., Irvine, CA), pupillary responses, including pupil size or light reflex, were measured every 2 hours during ICU stay. The light reflex was acquired using a flash of visible white light with an 800-ms duration at the start of each 3.2-second scan. The device automatically measures multiple parameters of pupil response, including resting pupil size, minimum pupil size after light stimulation, and constriction velocity.^[6]^

### 2.4. Follow-up and outcomes

**Figure 1.**
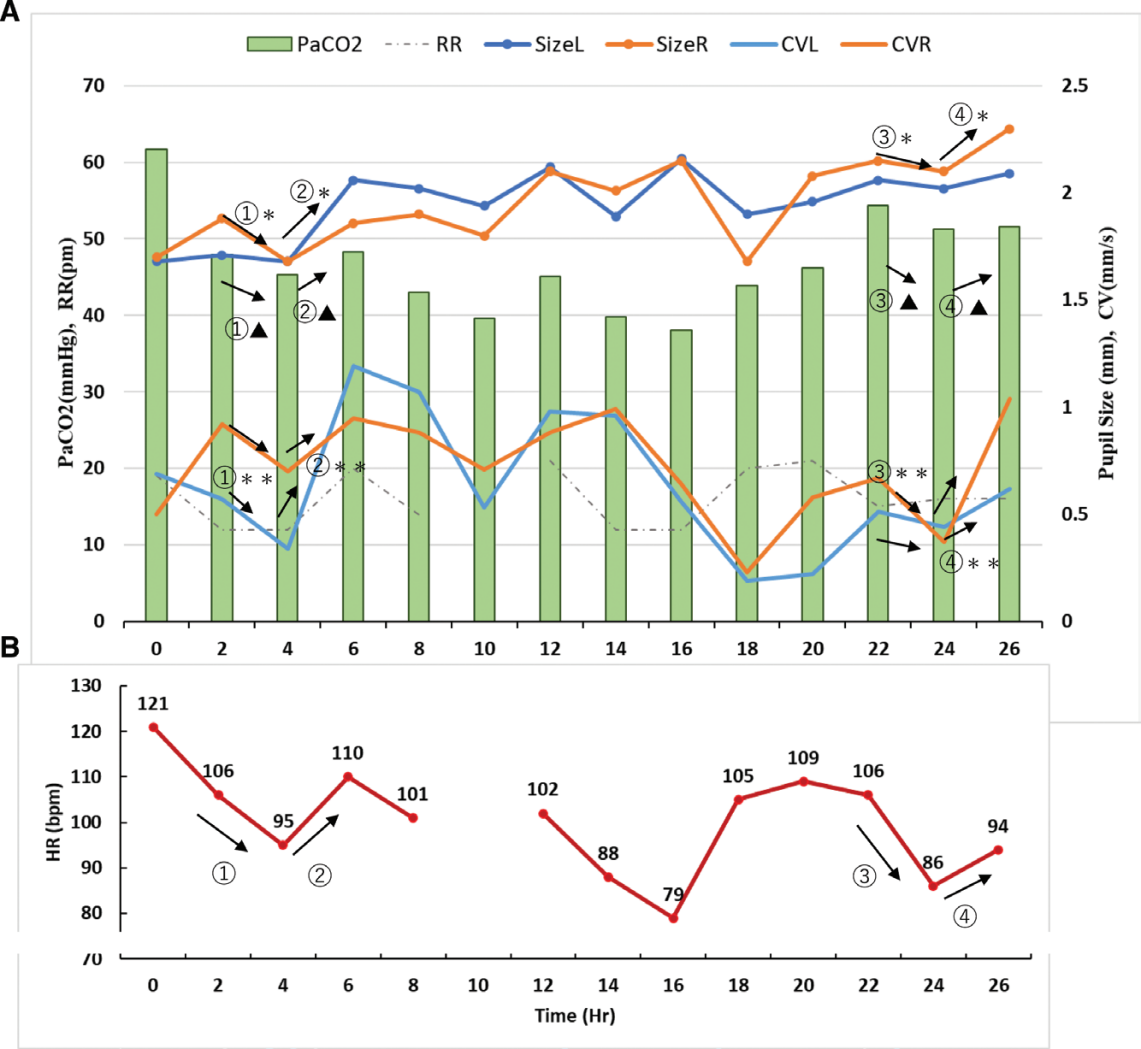
Relationships between sequential HR, RR, and PCO_2_ and pupillary response measured using a portable infrared quantitative pupilometer in CO_2_ narcosis. (A) Time course of pupillary size change and contraction velocity measured using automated pupillometry and their relationship with RR and PCO_2_, which is calculated as the amount of constriction divided by the duration of the constriction that shows the average velocity. Changes in HR correlated with pupil size. Tachycardia and hypercapnia were sustained in the CO_2_ narcosis case. Changes in HR correlated with pupil size and the mean contraction velocity. Increases or decreases in HR (①–④), pupil size (①*–④*), and mean construction velocity (①**–④**) were parallel. Changes in these parameters indicated autonomic nervous activity. Changes in PCO_2_ and RR correlated with pupil contraction velocity. Changes in PCO_2_ and RR, which regulate minute tidal volume, correlated with pupil size and mean CV. Increases or decreases in PCO_2_ (①▲, ③▲) (② ▲, ④ ▲), RR, pupil size (①*,③*)(②*,④*), and mean contraction velocity (①**,③**)(②**,④**) were parallel. CO_2_ = carbon dioxide, CV = constriction velocity (mm/s), HR = heart rate (bpm), PCO_2_ = partial pressure of carbon dioxide, RR (a broken line) = respiratory rate (per minute), Size L = pupil size on the left (mm), Size R = pupil size on the right (mm). (B) Changes of HR in a patient with CO_2_ narcosis.

The relationships between sequential HR, RR, and PCO_2_ values and the change in pupil size or pupillary response determined using a portable infrared quantitative pupilometer are shown in Figure 1A and B. Changes in RR and PCO_2_ values correlated with pupil size and constriction velocity. The increases or decreases in PCO_2_, RR, pupil size, or mean contraction velocity were parallel. By contrast, HR changes were contrary. Increases or decreases in HR, pupil size, and mean construction velocity were parallel, and changes in these parameters indicated AN activity.

One hour after intubation at ER, the patient’s impaired consciousness improved. The patient was extubated 20 hours later after admission to the ICU. Imaging, sputum culture, and blood culture were performed to detect the organisms responsible for pneumonia and other infections that could cause exacerbation of CO_2_ narcosis; however, the causative pathogen could not be identified. The patient improved quickly and was discharged from our hospital on Day 3 without any medical complications.

## 3. Discussion

Oxygen-induced hypercapnia can develop in some patients with COPD, and these patients depend on the hypoxemic respiratory drive due to attenuation of CO_2_ sensitivity. CO_2_ accumulation induces symptoms of central nervous system abnormalities, such as headache or altered consciousness.^[7]^

TV and ventilatory CO_2_ are useful indicators of AN activity,^[4,5]^ similar to blood pressure and HR.^[4]^ On the contrary, pupils are regulated by the AN.^[8]^ Miosis is observed in patients with severe narcosis and is considered a compensatory mechanism that occurs in response to initial sympathetic nervous activity. If the patient develops hypoxia, tachycardia, or tachypnea due to stimulation of the sympathetic nervous system, the patient finally enters a miotic state.

A portable automated pupilometer facilitates easy and accurate pupillary measurement compared to a penlight and does not depend on an evaluator’s skills. Furthermore, miosis has been observed in many patients in ICU as sedatives are often administered to critically ill patients during ICU treatment. Regardless of whether pupillary responses are influenced by these drugs, the values measured by infrared pupillometry are less influenced by the pharmacological effect of such drugs.^[9–11]^

The effectiveness of HRV monitoring in assessing autonomic response activity in severely ill patients in the ICU is suggested.^[1,2]^ However, HRV monitoring can be difficult to assess with precision for patients with tachycardia or arrhythmias. The pupillary response measured using an automated pupilometer is less influenced by the HR range.^[12]^ Several reports have suggested that pupillary size measurement is an easy-to-use tool for evaluating AN activity in clinical situations.^[13]^

In fact, measuring pupillary size may be the easiest approach to monitoring autonomic nervous responses in the clinic. AN activity affects not only pupillary size but also other pupillary responses through mechanisms of regulation of the pupillary response. However, other pupillary responses might be more sensitive than pupillary size to assess AN activity. For example, another study stated that pupillary oscillations correspond to the respiratory rhythm.^[14]^ Automated pupillary monitors can measure and monitor multiple pupillary parameters over time. To date, they have been used primarily for patients in ICU with central nervous system diseases.^[3]^ AN activity in patients with CO_2_ narcosis without AN dysfunction can be precisely assessed based on their pupillary response using a portable infrared quantitative pupilometer, as in this case, especially in patients with severe CO_2_ narcosis who have miosis, which is difficult to assess using penlights to determine subtle changes.

Studies including larger sample sizes in patients intoxicated with narcotics and other critically ill patients are required before validation can be established for comprehensive monitoring of autonomic nerve responses using portable automated pupillometry.

## 4. Conclusion

Portable automated pupillometry can measure the pupillary response easily and precisely, even in the presence of miosis. Data obtained using automated pupillometry might facilitate AN activity monitoring in critically ill patients in the ICU as small and lightweight medical devices are easier to handle. Given the preliminary nature of this study, more research is warranted to assess its potential value.

## Acknowledgments

The authors appreciate the patient’s consent to present this case.

## Authors contributions

**Conceptualization:** Junko Yamaguchi.

**Data curation:** Toru Hosokawa, Shingo Ihara.

**Investigation:** Junko Yamaguchi, Toru Hosokawa.

**Project administration:** Junko Yamaguchi.

**Supervision:** Kosaku Kinoshita.

**Visualization:** Junko Yamaguchi, Shingo Ihara.

**Writing – original draft:** Junko Yamaguchi.

**Writing – review & editing:** Junko Yamaguchi, Kosaku Kinoshita.
